# Editorial: Recent advances in insect olfaction: characterization of neural circuits from sensory input to motor output

**DOI:** 10.3389/fncel.2023.1282499

**Published:** 2023-09-05

**Authors:** Xin-Cheng Zhao, Bente G. Berg, Guirong Wang

**Affiliations:** ^1^Henan International Joint Laboratory of Green Pest Control, College of Plant Protection, Henan Agricultural University, Zhengzhou, China; ^2^Chemosensory Laboratory, Department of Psychology, Norwegian University of Science and Technology, Trondheim, Norway; ^3^Agricultural Genomics Institute at Shenzhen, Chinese Academy of Agricultural Sciences, Shenzhen, China

**Keywords:** antennal sensilla, palpal sensilla, primary olfactory center, olfactory circuit, neuromodulators

Insect olfaction plays an essential role in mediating various behaviors, for instance, locating a relevant host, mate, and oviposition site, as well as avoiding predators and toxic food. Studying the chemosensory circuits of insects is a suitable approach for understanding how olfactory information turns into behavior. The central objective of this Research Topic was to promote exploration of the neural pathways underlying olfaction in a wide range of insect species. The five original research papers and one review under this topic covered specific issues in a variety of insect species, including locusts, beetles, butterflies, aphids, fruit fly, and ants. These organisms seem to share a common sensory logic but have developed specialized adaptations to distinct ecological niches. Such research advances provide a solid basis for understanding the neural circuits underlying odor-driven behavior.

The original research paper by Wang et al. identified chemoreceptor genes in two butterfly species of the same family, *Pieris brassicae* and *Pieris rapae*. By using both genomic and transcriptomic analysis, the authors obtained new data that contributes to improved knowledge about the genes controlling olfactory sensory neurons. In addition, the cohabiting *Pieris* butterflies are good models for investigating the putative role of chemosensory genes in formation of sympatric species, meaning speciation in the absence of geographical constraint. Interestingly, the two sympatric *Pieris* butterfly species have, to a large extent, conserved the chemoreceptor genes regarding numbers, sequence identities, motifs, and structures. The ecological differences between these two butterflies might result from a quantitative shift in the expression of orthologous genes.

The original paper by Yang et al. includes a comparative study of morphological and physiological characteristics of olfactory sensilla on the antennae of three related aphid species. By using scanning electron microscopy, the authors found that their antennae are different in shape and length, but similar in respect to morphological types of sensilla. They all include placoid sensilla, coeloconic sensilla, and trichoid sensilla. The first two sensillum types, the placoid and coeloconic, were distributed on the antennal segments forming the primary rhinaria and the third type, the trichoid, was located on the secondary rhinaria. By utilizing the single sensillum recording technique, it was shown that the placoid and coeloconic sensilla detect plant-derived volatiles while the trichoid ones recognize sex pheromone components. Even though the primary rhinaria of the three aphid species showed a common functional role in terms of detecting plant odors, some species-specific responses to the plant volatiles were recorded. The physiological divergence of olfactory receptor neurons in the primary rhinaria of aphids clarifies peripheral mechanisms of olfactory recognition in closely related species. Such results also suggest how species-specific ecological niches are formed by olfactory behaviors.

The original paper by Watanabe et al. demonstrates how an eusocial insect, the Japanese carptenter ant, *Camponotus japonicas*, can recognize subtle differences in colony-specific sets of cuticular hydrocarbons (CHC). This capacity is linked to assemblies of olfactory sensory neurons (OSNs) housed inside antennal basiconic sensilla. The large number of co-housed OSNs within one single basiconic sensillum, i.e., 140, proves the complexity of the peripheral encoding of odorants in these ants. Electrophysiological recordings from single sensilla showed, firstly, that application of each of 18 CHC activated different sets of OSNs, and, secondly, that each neuron was broadly tuned to CHCs. Besides, the authors found that multiple olfactory sensory neurons co-located within one sensillum were electrically coupled and fired in synchrony. Furthermore, ants from different colonies and those from different breeding groups of the same colony exhibited sensory neurons displaying distinct CHC response spectra as well. These results indicate that the olfactory sensory neurons of these ants utilize combinatorial coding for discriminating between nestmates and non-nestmates.

The original paper by Lv et al. deals with the detection of serotonin and GABA in the peripheral part of the olfactory system in the locust *Locusta migratoria*. Briefly explained, the authors found one relevant receptor type for each of the two well-known neurotransmitters by using *in situ* hybridization; the Lmig5-HT2 receptor was located in the accessory cells, while the LmigGABAb receptor was found in the olfactory sensory neurons. Knocking down the two receptors and performing consecutive single-unit electrophysiological recordings combined with RNA interference experiments led to significantly higher responses in mutant than in non-mutant locusts.

The original paper by Trebels et al. follow up a previous publication describing the palpal division of the olfactory system in the red flour beetle *Tribolium castaneum* (Dippel et al., 2016). In this holometabolous insect, the olfactory sensory neurons (OSNs) projecting from the palps terminate in distinct regions outside the antennal lobe—differently from the palpal projection pattern in other holometabolous species, which is confined to the antennal lobe—together with the antennal OSNs. Interestingly, the separation of projection terminals from the antenna and palps, as reported in the red flour beetle, is more like the arrangement in hemimetabolous insects. The results of the study performed here, include (1) a confirmation of the formerly described palpal projection pattern including OSN axons terminating in the paired lobus glomerulatus (LG) and the unpaired gnathal olfactory center (GOC), (2) identification of the chemosensory sensillum categories and an estimate of their distribution on the two types of palps, (3) description of the GOC glomerular organization by means of a 3D-reconstruction, and (4) a description of the distribution of an assembly of relevant neurochemicals (GABA/GAD, allatotropin, tachykinin-related peptides, myoinhibitory peptides, and serotonin), obtained by utilizing the immunostaining technique.

The article by Fabian and Sachse is a review on experience-dependent plasticity in the olfactory system of the fruit fly and other insects. Here, the authors discuss not only common problems related to the study of this issue, but also ways to overcome the challenges and develop future directions in this area of science. As pointed out by Fabian and Sachse, there was no review of recent findings in this research field. To clarify which phenomena should be referred to as plasticity, the authors summarized the historical establishment of this term in neuroscience. Recent advances in the study of experience-dependent plasticity effects in the olfactory system of *Drosophila melanogaster* were given particular attention due to its distinctive accessibility related to use of genetic tools. In addition to recent findings obtained in the fruit fly, effects observed in Hymenoptera, Lepidoptera, and Orthoptera were addressed as well. Investigation of mechanisms that allow organisms to adapt to new environmental conditions, i.e., experience-dependent plasticity, is a crucial topic for future research, especially in an area of rapid environmental changes.

In addition to the peripheral olfactory sensilla and the primary olfactory center, higher olfactory areas initiating motor output play a vital role in turning odor information into behavioral responses. Unfortunately, no papers concerning such issues were collected on this topic. Investigation of the higher olfactory circuits should receive more attention in the future. Exploring the processing principles of all levels of the olfactory pathway is of essential importance for understanding the odor code.

## Author contributions

X-CZ: Funding acquisition, Writing—original draft, Writing—review and editing. BB: Funding acquisition, Writing—original draft, Writing—review and editing. GW: Funding acquisition, Writing—original draft, Writing—review and editing.
